# Characterization and Antibacterial Activity of a Polysaccharide Produced From Sugarcane Molasses by *Chaetomium globosum* CGMCC 6882

**DOI:** 10.3389/fnut.2022.935632

**Published:** 2022-06-21

**Authors:** Li Ma, Xueliang Guo, Jiaoyang Yang, Xiangru Zeng, Kaili Ma, Lu Wang, Qi Sun, Zichao Wang

**Affiliations:** ^1^Henan Provincial Key University Laboratory for Plant-Microbe Interactions, College of Biology and Food, Shangqiu Normal University, Shangqiu, China; ^2^School of Biological Engineering, Henan University of Technology, Zhengzhou, China; ^3^College of Life Sciences, Chongqing Normal University, Chongqing, China; ^4^National Engineering Laboratory, Key Laboratory of Henan Province, Henan University of Technology, Zhengzhou, China

**Keywords:** sugarcane molasses, *Chaetomium globosum* CGMCC 6882, polysaccharide, structural characteristics, antibacterial activity

## Abstract

As a by-product of the sugar industry containing many sugars, proteins, nitrogenous materials, and heavy metals, molasses is rarely used for polysaccharide production. In the present work, a *Chaetomium globosum* CGMCC 6882 polysaccharide was produced from sugarcane molasses (CGP-SM) was successfully produced from sugarcane molasses. The yield of CGP-SM was 5.83 ± 0.09 g/l and its protein content was 2.41 ± 0.12% (w/w). Structural analysis showed that CGP-SM was a crystalline and amorphous polysaccharide containing rhamnose, glucosamine, galactose, glucose, mannose, fructose, and glucuronic acid in the molar ratio of 10.31: 1.14: 2.07: 59.55: 42.65: 1.92: 9.63. Meanwhile, weight-average molecular weight (Mw), number-average molecular weight (Mn), and polydispersity (Mw/Mn) of CGP-SM were 28.37 KDa, 23.66 KDa, and 1.199, respectively. Furthermore, the bacteriostatic assay indicated that CGP-SM inhibited the growth of *Escherichia coli* and *Staphylococcus aureus* in a concentration-dependent manner, and its inhibitory effect on *S. aureus* was higher than that of *E. coli*. Above all, this work provides a green method for the production of bioactive polysaccharide from sugarcane molasses.

## Introduction

Microbial polysaccharides, such as lipopolysaccharides (LPS), capsular polysaccharides (CPS), and exo-polysaccharides (EPS), are polymers of biological carbohydrates with high molecular weight and constitute an important part of renewable polymer resources (1). CPS is mainly associated with pathogenicity and toxin promoters in bacteria, LPS is present in the outer membrane of bacteria and important for human immune response, and EPS is a bacterial extracellular polymer (2). EPS usually consists of monosaccharide and non-carbohydrate substituent, which can be divided into two types according to the composition of sugar units, one is a homologous polysaccharide composed of only one monosaccharide unit (3), and the other is heteropolysaccharide containing two or more monosaccharide units (4). EPS not only has superior rheological properties, bio-adhesion, non-toxicity, biocompatibility, and biodegradation (5, 6), but also can be produced from renewable resources and waste (7–9), which makes it popular in the food and other industries.

Molasses is the main accessory substance in the sugar manufacturing process, which contains about 50% (w/w) sugars, 1.0% (w/w) nitrogenous materials, and heavy metals (such as Fe^2+^, Cu^2+^, Mn^2+^, Mg^2+^, Ca^2+^, etc.) (10). The total yearly yield of molasses is about 55 million tons worldwide, but it is simply discharged or applied to feed due to its thick, semi-flowing, and dark brown properties (11). With the rapid development of microbial fermentation and bio-catalysis transformation technologies in recent years, more and more value-added bio-products have been produced from molasses *via* microbial transformation, such as bio-fuels, enzymes, organic acids, etc. (12). Meanwhile, many polysaccharides have also been produced from molasses, including pullulan (13), welan gum (14), hyaluronic acid (4), and glucan (3). The above research provided help for the production of polysaccharides from low-cost molasses with an environmentally microbial process.

As an endophytic fungus isolated from *Gynostemma pentaphyllum* herb, *Chaetomium globosum* CGMCC 6882 could use many kinds of processing wastes as a carbon source to produce polysaccharides *via* submerged fermentation. For instance, crude glycerol could be used by *C. globosum* CGMCC 6882 to produce anticancer polysaccharides (15). At the same time, *C. globosum* CGMCC 6882 could use wheat straw and distillers’ grain to produce antioxidant and anti-inflammatory polysaccharides (7, 9). However, the utilization of molasses for polysaccharide production by *C. globosum* CGMCC 6882 was neglected up to now. Therefore, a polysaccharide of CGP-SM was produced from sugarcane molasses by *C. globosum* CGMCC 6882 with submerged cultivation presently. On the one hand, the physicochemical properties of CGP-SM were characterized. On the other hand, the inhibition effects of CGP-SM against *Escherichia coli* and *Staphylococcus aureus* were assayed. We hope that this work could provide help for further developing the scope of bioactive polysaccharide production by microorganisms from molasses.

## Materials and Methods

### Materials and Chemicals

Sugarcane molasses was purchased from Xinze biological Co. Ltd. (Zhengzhou, China), and contained 8% (w/w) glucose, 28% (w/w) sucrose, 2.1% (w/w) other carbohydrates, 12% (w/w) fructose, 3.6% (w/w) crude protein, 0.05% (w/w) crude fat, 8.2% (w/w) ash, 5.3% (w/w) salt, and 7.5% (w/w) metal ions. Rhamnose, fucose, fructose, galactose, glucose, glucosamine, xylose, mannose, arabinose, galacturonic acid, and glucuronic acid used in the present work were purchased from Sigma-Aldrich (Shanghai, China). Meanwhile, other chemical reagents were bought from Sinopharm Chemical Reagent Co., Ltd. (Shanghai, China).

### Organisms

*Chaetomium globosum* CGMCC 6882 was stored in China General Microbiological Culture Collection Center (China). *E. coli* and *S. aureus* were bought from the China Center of Industrial Culture Collection (CICC), the collection numbers were CICC10899 and CICC10001, respectively.

### Pre-treatment of Sugarcane Molasses

Pre-treatment of sugarcane molasses was based on the methods reported previously by Ai et al. (16) with some modifications. Briefly, crude sugarcane molasses was diluted by adding four folds (w/w) deionized water and filtered *via* a 0.45 μm drainage membrane filter (Beijing Solarbio Science & Technology Co., Ltd., Beijing, China). The filtrate was heated for 30 min at 100°C and centrifuged (TGL-16M, Xiangyi Centrifuge Instrument Co., Ltd.) at 10,000 × *g* for 15 min, then the supernatant was collected for further use.

### Polysaccharide Production From Sugarcane Molasses

The fermentation medium used for CGP-SM production by *C. globosum* CGMCC 6882 contained only 40 g/l above the sugarcane molasses supernatant. Batch fermentation was carried out in a 7.0 L fermenter (BioFlo 115, New Brunswick, United States) at 28°C with 3.5 L medium, cultivation lasted for 7 days, inoculation volume was 5% (v/v), culture pH was kept between 6.80 and 7.20 with the addition of 4 mol/l NaOH and 4 mol/l HCl, the agitation was 100 rpm and the aeration was 0.8 vvm. CGP-SM extraction and purification were conducted according to the methods reported previously (17) with some modifications. After fermentation, the fermented liquid was filtered and centrifuged to remove impurities. Then the broth was concentrated at 60°C and 0.1 MPa. After that, the concentrated broth was de-proteinized by adding three volumes of Sevag solution. Finally, three volumes of cold alcohol was added and kept at 4°C overnight to precipitate CGP-SM, the precipitated CGP-SM was washed three times with 75% cold alcohol and lyophilized to obtain crude CGP-SM. The crude CGP-SM was re-dissolved in distilled water and de-pigmented with AB-8 macroporous resin. Then, the CGP-SM solution was dialyzed for 48 h in distilled water (M_*W*_ cut-off was 10 kDa). After that, the CGP-SM solution was filtered through a 0.22 μm filter and applied to a Sepharose CL-6B column (2.5 × 60 cm) for further purification, and eluted with 0.1 mol/l NaCl at a flow rate of 0.6 ml/min. In the end, the fraction was collected and freeze-dried for the following analysis.

### Physicochemical Properties Analysis

#### Determination of Monosaccharide Composition and Protein Content

Protein content in CGP-SM was detected by the Coomassie Brilliant Blue method with bovine serum albumin as standard (18). Meanwhile, CGP-SM was dissolved in 2 mol/l trifluoroacetic acid (TFA) and hydrolyzed at 120°C for 2 h, then the hydrolysate was washed three times with methanol and evaporated to dryness for removing TFA. The hydrolyzed material was transferred into a 25 ml volumetric flask, diluted to 25 ml by adding deionized water, and detected using high-performance anion-exchange chromatography (HPAEC) equipped with Dionex ICS5000 system (Dionex, United States) and CarboPac PA20 column (ID 3 mm × 150 mm) as reported previously (19).

#### Determination of Molecular Weight

CGP-SM was dissolved in 0.1 mol/l NaNO_3_ and filtered by a 0.5 μm microfiltration membrane. Then, the molecular weight of CGP-SM was detected by high-performance gel filtration chromatography (HPGFC) equipped with a refractive index detector and a Superose 12 column (1.0 cm × 30.0 cm) as the method reported by Li et al. (20) with some modifications.

#### Fourier Transform Infrared Spectroscopic Analysis

A Nexus 470 Fourier transform infrared (FT-IR) spectrometer (Nicolet, United States) was used to detect the FT-IR spectra of CGP-SM. Briefly, 5 mg CGP-SM was fully ground with 1 g KBr and pressed to a pallet for FT-IR spectra determination between 4,000 and 400 cm^–1^ as reported previously (17).

#### Nuclear Magnetic Resonance Analysis

The nuclear magnetic resonance (NMR) spectra of CGP-SM were recorded by a Bruker Avance 500 MHz spectrometer (Bruker Inc., Germany). Briefly, CGP-SM was dissolved in a 5 mm NMR tube with 1 ml D_2_O and processed by ultrasonic treatment for 30 min as reported previously (19). Then, the ^1^H NMR and ^13^C NMR spectra of CGP-SM were recorded in parts per million.

#### Scanning Electron Microscopy Observation

Morphological images of CGP-SM were recorded by a Quant 200 scanning electron microscopy (SEM) (FEI, Netherlands) as reported by Yan et al. (21) with some modifications. Briefly, the freeze-dried CGP-SM sample was fixed onto a metal observation stage placed in a vacuum, and the accelerating voltage was 15 kV.

#### X-Ray Diffraction Analysis

A D8advance X-ray diffractometer (Bruker, Germany) was used to analyze the crystallinity of CGP-SM as reported previously with some modifications (22). Cu-Kα was used as the radiation source, the scanning angle range was set as 10–60° (2θ). The scanning voltage was 30 kV and the scanning current was 30 mA. Meanwhile, the scanning rate was 2°/min and the step size was 0.02°.

### Antibacterial Activity Assay

Inhibitory effects of CGP-SM against *E. coli* and *S. aureus* were analyzed by using the inhibition zone method as reported previously (23) with some modifications. Briefly, CGP-SM was dissolved in deionized water to 0.125, 0.25, 0.5, 1.0, and 2.0 mg/ml, respectively, filtrated through a 0.22 μm drainage membrane filter (Beijing Solarbio Science & Technology Co., Ltd.). Then, nutrient agar (20 ml) was added into plates and solidified, after which, 150 μl test organism (10^6^ CFU/ml) suspension was spread on the agar plate surface. Then, one sterilized 2 mm circular paper was placed onto the middle of the plate, and 10 μl of CGP-SM samples were injected onto the circular paper. The inhibition zone of each plate was measured by diameter after incubation at 37 ± 1°C for 24 h. Antibacterial activities of CGP-SM against *E. coli* and *S. aureus* were analyzed by determining the diameters of inhibition zones with a Vernier caliper.

### Statistical Analysis

Data were expressed as means ± SD after triplicate repeats. Data were subjected to one-way ANOVA, and the significant differences were analyzed using SPSS version 19.0 (IBM, United States).

## Results and Discussion

### Polysaccharide Production

**FIGURE 1:**
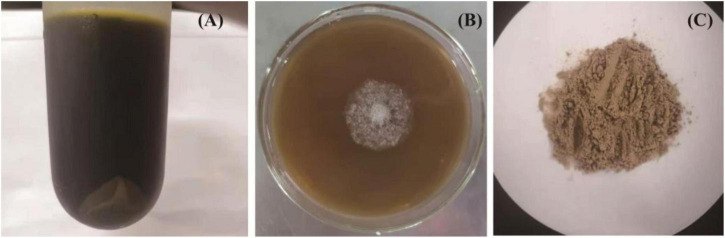
The distilled, heated, and centrifuged sugarcane molasses **(A)**, growth profile of *Chaetomium globosum* CGMCC 6882 on sugarcane molasses plate **(B)**, polysaccharide (CGP-SM) powder obtained from submerged fermentation **(C)**.

In the present work, after sugarcane molasses was distilled, heated, and centrifuged, the supernatant (Figure 1A) could be used as the only carbon source for *C. globosum* CGMCC 6882 growth (Figure 1B), which demonstrated that sugarcane molasses is a promising substrate for metabolites production by *C. globosum* CGMCC 6882. Meanwhile, after submerged cultivation, extraction, and purification, a polysaccharide (CGP-SM) was obtained (Figure 1C) and its yield was 5.83 ± 0.09 g/l (Table 1), the color of CGP-SM might be due to the pigments in which it was left. This yield is lower than welan gum (37.65 g/l) (14) and sophorolipid (53 ± 3 g/l) (24) produced from molasses, but higher than chitosan (0.39 g/l) (25) and hyaluronic acid (3.48 g/l) (4) produced from molasses with submerged cultivation. Many factors, such as microbial species, culture conditions, and nutritional types, could affect polysaccharide yields (26), but submerged cultivation has the advantages of better controlling cultivation conditions, less pollution, less time consumption, high efficiency, and less space occupation. Meanwhile, Table 1 showed that CGP-SM contained 2.41 ± 0.12% (w/w) protein, which might be due to the unused protein in sugarcane molasses bonded to CGP-SM during the extraction process.

**TABLE 1:** Characteristics of polysaccharide produced by *Chaetomium globosum* CGMCC 6882 from sugarcane molasses.

Parameters	CGP-SM
Polysaccharide yield (g/L)	5.83 ± 0.09
Protein content (w%)	2.41 ± 0.12
Monosaccharide composition (μmol/L)	
Rhamnose	10.31
Glucosamine	1.14
Galactose	2.07
Glucose	59.55
Mannose	42.65
Fructose	1.92
Glucuronic acid	9.63
Molecular weight (1 × 10^3^ Da)	
Weight-average molecular weight (M_*w*_)	28.37
Number-average molecular weight (M_*n*_)	23.66
Polydispersity (M_*w*_/M_*n*_)	1.199

### Physicochemical Properties of CGP-SM

#### Monosaccharide Composition

Except for polysaccharide source, extraction, and purification methods, monosaccharide diversity of microbial exo-polysaccharide is mainly affected by the culture conditions and culture medium nutrients (27). As shown in Table 1 and Supplementary Figure 1, the monosaccharide composition of CGP-SM was rhamnose, glucosamine, galactose, glucose, mannose, fructose, and glucuronic acid in the molar ratio of 10.31: 1.14: 2.07: 59.55: 42.65: 1.92: 9.63. Previously, when wheat straw was used as the only carbon source, the monosaccharide composition of polysaccharide produced by *C. globosum* CGMCC 6882 was rhamnose, glucosamine, galactose, glucose, xylose, fructose, and glucuronic acid in the molar ratio of 21.46: 1.58: 1.11: 55.15: 36.37: 7.04: 7.34 (9). When glucose was used as the only carbon source, the monosaccharide composition of polysaccharide produced by *C. globosum* CGMCC 6882 was arabinose, galactose, glucose, xylose, mannose, and glucuronic acid in the molar ratio of 0.64: 2.58: 23.53: 0.90: 2.47: 0.27 (19). At the same time, monosaccharide composition of polysaccharide produced by *C. globosum* CGMCC 6882 was rhamnose, arabinose, galactose, glucose, xylose, mannose, galacturonic acid, and glucuronic acid in the molar ratio of 4.11: 7.34: 13.31: 20.99: 1.07: 0.91: 4.75: 0.36 with distillers’ grain as the only carbon source (7). However, monosaccharide compositions of polysaccharides produced by *C. globosum* CGMCC 6882 were galactose, glucose, mannose, and glucuronic acid in molar ratios of 5.95: 58.75: 5.65: 0.76 and 8.16: 43.77: 5.84: 0.43 with glycerol and crude glycerol were used as carbon source, respectively (15).

#### Molecular Weight

As shown in Table 1, the weight-average molecular weight (Mw) and number-average molecular weight (Mn) of CGP-SM were 28.37 and 23.66 KDa, respectively, and its polydispersity (Mw/Mn) was 1.199. Previously, Zhang et al. (28) found that molecular weight could affect the antibacterial activities of polysaccharides produced by *C. globosum* CGMCC 6882, and the lower molecular weight endowed polysaccharide with higher antibacterial activity. Meanwhile, Zheng et al. (29) found that the antioxidant activity of polysaccharide produced by *Pholiota nameko* PN-01 was enhanced with the decrease in its molecular weight. However, Dou et al. (30) reported that the antioxidant and α-glucosidase inhibitory activities of blackberry fruit polysaccharides were coincident with their molecular weights. Furthermore, Cai et al. (31) demonstrated that *Ganoderma lucidum* polysaccharide had higher anti-fatigue activity when its molecular weight was higher than 10 kDa.

#### Fourier-Transform Infrared Spectra

**FIGURE 2:**
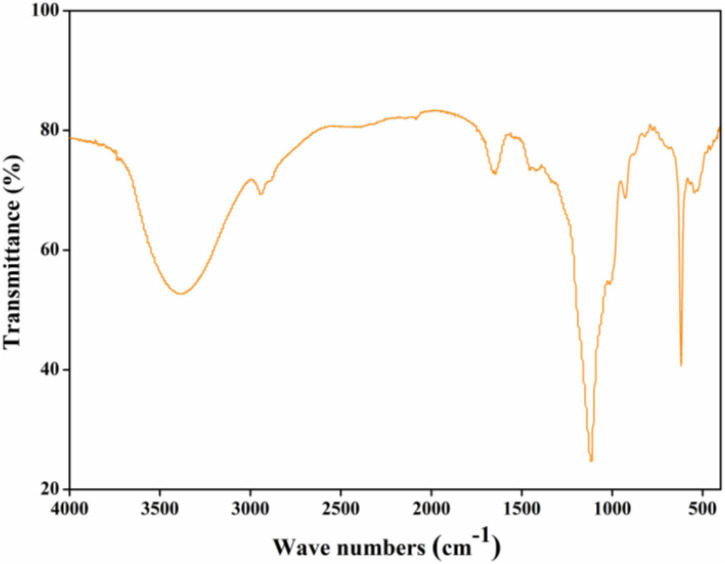
Fourier-transform infrared (FT-IR) spectrum of CGP-SM.

As can be seen from Figure 2, a broad peak at around 3,400 cm^–1^ might relate to the O-H stretching vibration of intra-molecular or inter-molecular hydrogen bonds (19). A typical peak at around 2,900 cm^–1^ might attribute to C-H tensile vibration such as CH, CH_2_, and CH_3_ groups, the absorption peak at around 2,400 cm^–1^ might relate to aliphatic C-H bonds stretching (7). The absorption peaks at around 1,700 and 1,250 cm^–1^ might be derived from C=O stretching vibration in the ester or carboxyl groups, and absorption peak at around 1,600 cm^–1^ might be induced by the symmetrical C=O stretching vibrations in carboxyl groups (9). The absorption peak at around 1,400 cm^–1^ might correspond to asymmetrical C=O stretching vibrations coupled with C-H bending vibrations, and absorption peaks between 1,000 and 1,200 cm^–1^ might assign to C-O-H and C-O-C stretching vibrations. Absorption peaks between 900 and 800 cm^–1^ might assign to the β-glycosidic bonds and α-type glycosidic linkages in CGP-SM.

#### Nuclear Magnetic Resonance Spectra

**FIGURE 3:**
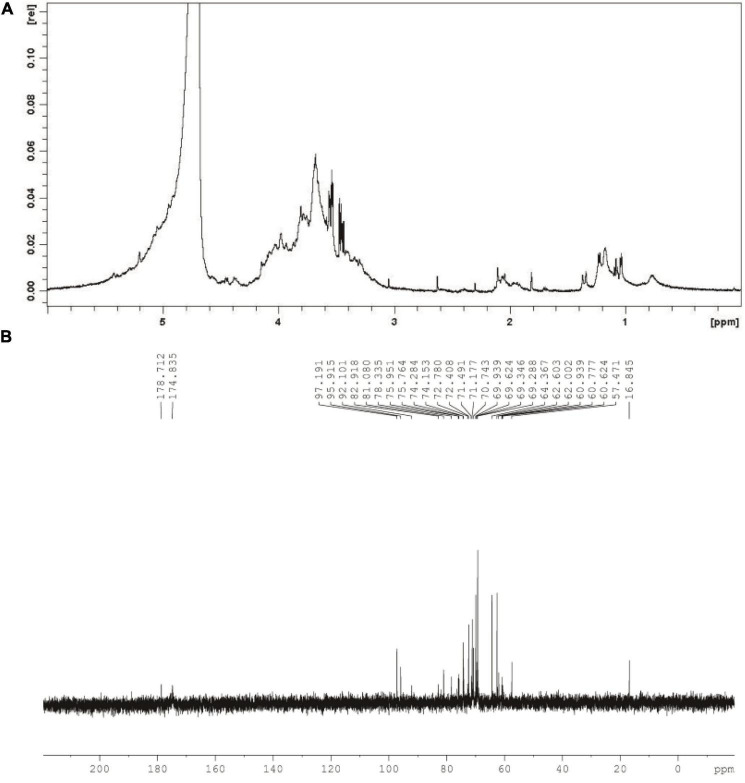
^1^H nuclear magnetic resonance (NMR) spectrum **(A)** and ^13^C NMR spectrum **(B)** of CGP-SM.

As can be seen from Figure 3A, chemical shift at around 4.8 ppm might assign to D_2_O used in the present work. Meanwhile, singles between 4.0 and 3.4 ppm might relate to glycosyl residues in CGP-SM. The ^1^H signal at approximately 2.0 ppm might relate to the *O*-acetyl groups in CGP-SM. Furthermore, singles at around 1.2 ppm might be due to the proton of the CH_3_ group in Rha*p* residues (32). In addition, the ^13^C NMR spectrum of CGP-SM showed several anomeric carbon signals ranging from 0.0 to 200.0 ppm (Figure 3B). The spectrum signals at around 175 and 180 ppm might relate to the ionic carboxyl group in uronic acid (33). The signal peaks at around 96 and 97 ppm might relate to anomeric carbons in Glc*p* and Ara*p*. The signals observed at around 60 and 80 ppm in the anomeric region of the ^13^C NMR spectra might assign to the C-2, C-3, C-4, C-5, and C-6 of Glc*p* (34). Signal peak at around 16.8 ppm might relate to C-6 in Rha*p*.

#### Scanning Electron Microscopy

**FIGURE 4:**
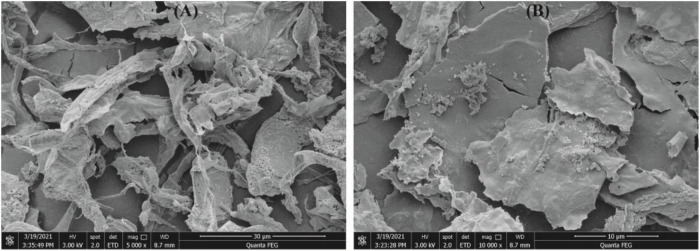
Scanning electron microscopy (SEM) photographs of CGP-SM with different magnifications. **(A)** ×5,000, **(B)** ×10,000.

Scanning electron microscopy (SEM) is an effective instrument to observe the morphological image, shape, and porosity of polysaccharides (35). As shown in Figure 4A, CGP-SM had irregular structures and ribbons with branches. Meanwhile, Figure 4B showed that CGP-SM exhibited an irregular, fibrous, and smooth surface, which might relate to the low content of uronic acid leading to few interaction points among particle (30). These results are comparable to the polysaccharide reported by López-Legarda et al. (36), but different from the polysaccharide reported by Xiong et al. (37). The shape, structure, and surface morphology of polysaccharides are not only affected by the processes of extraction, solubility, purification, and lyophilization but also influenced by the culture conditions (36).

#### X-Ray Diffractometry

**FIGURE 5:**
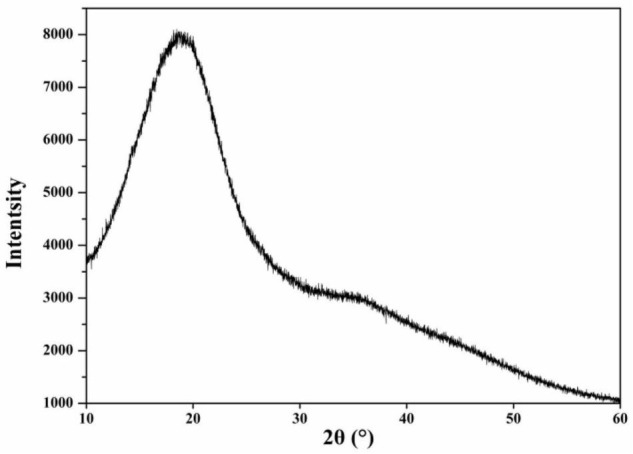
X-ray diffractogram of CGP-SM.

In the present work, the amorphous and crystalline information of CGP-SM were analyzed by an X-ray diffraction (XRD) and the results were shown in Figure 5. As can be seen from Figure 5, there was a broad diffraction peak appeared at around 20°, which indicated that CGP-SM was a crystalline and amorphous structure of polysaccharide. This structural feature was comparable to the *Fritillaria pallidiflora* Schrenk polysaccharide reported by Rozi et al. (38), but the XRD result of polysaccharide reported by Dou et al. (30) was different from the present work.

### Antibacterial Activity

**FIGURE 6:**
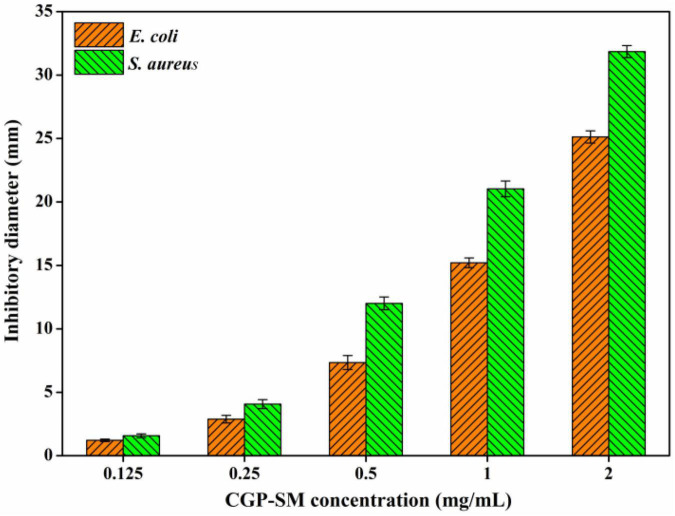
Antibacterial activity of CGP-SM against *Escherichia coli* and *Staphylococcus aureus* with different concentrations.

Bacteriostatic activities of CGP-SM against *E. coli* and *S. aureus* were analyzed by using inhibition zone diameter (mm) methods. Figure 6 showed that the inhibitory effects of CGP-SM against *E. coli* and *S. aureus* increased with the increase of its concentration from 0.125 to 2.0 mg/ml, and its inhibitory effect on *S. aureus* was higher than that of *E. coli*. When the concentration of CGP-SM was 0.125 mg/ml, inhibition zones of CGP-SM against *E. coli* and *S. aureus* were 1.21 ± 0.17 mm and 1.57 ± 0.13 mm, respectively. When its concentration reached 2.0 mg/ml, inhibition zones of CGP-SM against *E. coli* and *S. aureus* were 25.12 ± 0.48 mm and 31.85 ± 0.39 mm (Supplementary Figure 2), respectively. Meanwhile, researchers obtained comparable bacteriostatic activity of polysaccharides against *E. coli* and *S. aureus*. For instance, Qiu et al. (39) demonstrated that the antibacterial activity of β-glucan oligosaccharides against *S. aureus* and *E. coli* increased with the increase of its concentration, and its inhibitory effect on *S. aureus* was higher than that of *E. coli*. Meanwhile, Meng et al. (40) reported that a water-soluble *Diaphragma juglandis* polysaccharide had a higher inhibitory effect on *S. aureus* than *E. coli*. However, Jiang et al. (41) found that mung bean (*Vigna radiate*) skin polysaccharide had antibacterial activity against *S. aureus*, but not *E. coli*. There are many factors, such as environmental conditions, polysaccharide structure, and microbial species that could influence the bacteriostatic effect of polysaccharides, and the antibacterial mechanisms of different polysaccharides are also varied (42). Furthermore, the antibacterial mechanisms of CGP-SM against *S. aureus* and *E. coli* will be analyzed in future work.

## Conclusion

With the rapid development of microbial fermentation and biocatalysis transformation technology over the last decade, increasing numbers of value-added bio-products have been produced from molasses by microbial conversion. In the present work, an antibacterial polysaccharide of CGP-SM was produced from sugarcane molasses by *C. globosum* CGMCC 6882 with submerged cultivation, but its yield was only 5.83 ± 0.09 g/l. Although this work provides help for the production of bioactive polysaccharide from low-cost molasses with the environmentally microbial process, how to achieve the efficient production of bioactive polysaccharides through molasses pretreatment, microbial gene reconstruction, and fermentation process optimization will be the focus of future research.

## Data Availability Statement

The original contributions presented in this study are included in the article/Supplementary Material, further inquiries can be directed to the corresponding authors.

## Author Contributions

LM contributed to the conception, design, and funding of the study. XG, JY, XZ, and KM organized the database. LW wrote the first draft of the manuscript. QS and ZW contributed to the writing—review and editing. All authors contributed to the article and approved the submitted version.

## Conflict of Interest

The authors declare that the research was conducted in the absence of any commercial or financial relationships that could be construed as a potential conflict of interest.

## Publisher’s Note

All claims expressed in this article are solely those of the authors and do not necessarily represent those of their affiliated organizations, or those of the publisher, the editors and the reviewers. Any product that may be evaluated in this article, or claim that may be made by its manufacturer, is not guaranteed or endorsed by the publisher.
