# Supplementary material for: Characterization and Antibacterial Activity of a Polysaccharide Produced From Sugarcane Molasses by Chaetomium globosum CGMCC 6882
Source: Front Nutr. 2022 Jun 21;9:935632. doi: 10.3389/fnut.2022.935632 (PMC9254729; doi:10.3389/fnut.2022.935632)
Supplement: Supplementary file 1 [file Data_Sheet_1.doc]

**Fig. S1** High performance anion exchange chromatography profile of CGP-SM.

**
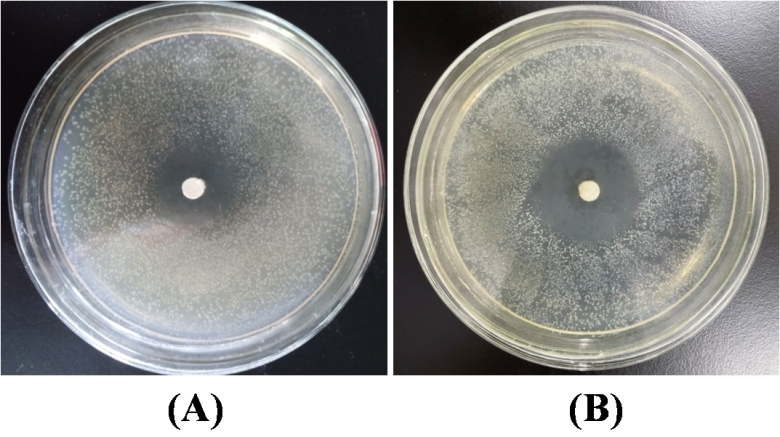
**

**Fig. S2** Inhibitory morphologies of CGP-SM against *E. coli* (A) and *S. aureus* (B).
